# Regulatory T Cell- and Natural Killer Cell-Mediated Inflammation, Cerebral Vasospasm, and Delayed Cerebral Ischemia in Aneurysmal Subarachnoid Hemorrhage—A Systematic Review and Meta-Analysis Approach

**DOI:** 10.3390/ijms26031276

**Published:** 2025-02-01

**Authors:** Andreas Pfnür, Benjamin Mayer, Lena Dörfer, Hayrettin Tumani, Daniel Spitzer, Markus Huber-Lang, Thomas Kapapa

**Affiliations:** 1 Department of Neurosurgery, University Hospital Ulm, Albert-Einstein-Allee 23, 89081 Ulm, Germany; 2 Institute of Epidemiology and Medical Biometry, University of Ulm, Helmholtzstr. 22, 89081 Ulm, Germany; 3 Institute for Clinical and Experimental Trauma Immunology, University Hospital Ulm, Helmholtzstr. 8/, 89081 Ulm, Germany; 4 Department of Neurology, University Hospital Ulm, Oberer Eselsberg 45, 89081 Ulm, Germany; 5 Department of Neurology, University Hospital Frankfurt, Theodor-Stern-Kai 7, 60596 Frankfurt am Main, Germany

**Keywords:** subarachnoid hemorrhage, natural killer cells, T cells, outcome, delayed cerebral ischemia, vasospasm

## Abstract

Aneurysmal subarachnoid hemorrhage (SAH) involves a significant influx of blood into the cerebrospinal fluid, representing a severe form of stroke. Despite advancements in aneurysm closure and neuro-intensive care, outcomes remain impaired due to cerebral vasospasm and delayed cerebral ischemia (DCI). Previous pharmacological therapies have not successfully reduced DCI while improving overall outcomes. As a result, significant efforts are underway to better understand the cellular and molecular mechanisms involved. This review focuses on the activation and effects of immune cells after SAH and their interactions with neurotoxic and vasoactive substances as well as inflammatory mediators. Particular attention is given to clinical studies highlighting the roles of natural killer (NK) cells and regulatory T cells (Treg) cells. Alongside microglia, astrocytes, and oligodendrocytes, NK cells and Treg cells are key contributors to the inflammatory cascade following SAH. Their involvement in modulating the neuro-inflammatory response, vasospasm, and DCI underscores their potential as therapeutic targets and prognostic markers in the post-SAH recovery process. We conducted a systematic review on T cell- and natural killer cell-mediated inflammation and their roles in cerebral vasospasm and delayed cerebral ischemia. We conducted a meta-analysis to evaluate outcomes and mortality in studies focused on NK cell- and T cell-mediated mechanisms.

## 1. Introduction

Aneurysmal subarachnoid hemorrhage (SAH) represents a life-threatening emergency that occurs with a variably reported incidence of between 4 and 23 individuals per 100,000, between 50 and 60 years of age, and more frequently in women (men/women = 1:1.1–1.6) [1,2]. This acute hemorrhagic CNS condition, which is present in approximately 2–7% of the group of strokes, is capable of causing severe morbidity and a high rate of mortality (8–70%) [1,3,4,5,6,7,8,9]. The morbidity rate is reported to be 4–31%, with moderately severe to severe disability on the modified Rankin Scale [10,11]. Since the 1990s, many factors have changed in the treatment of SAH, including technical improvements such as the introduction of high-resolution computed tomography angiography, developments in neuro-intensive care with the spread of specialized intensive care units, the standardized use of calcium channel blockers for the prophylaxis of cerebral vasospasm (CV) and delayed cerebral ischemia (DCI), and the introduction of and improvement in endovascular aneurysm occlusion methods. In summary, all these components could considerably reduce mortality after SAH by 17% [7,9,12]. In the constellation of technical and medical improvements addressing continuing challenges to improve outcomes, the incidence of CV and, in particular, DCI play a major role after SAH [13]. DCI is currently considered one of the most important clinical factors influencing short- and long-term outcomes, yet there are few effective and scientifically validated clinical approaches to its management [14].

CV as a pathologic, reversible narrowing of cerebral arteries, occurs angiographically in about 50–67% of all patients with SAH; however, only in 22–29% does it have unfavorable relevance to the clinical outcome [15,16,17,18]. The occurrence rate of DCI is described to be 25–28% [15,18]. However, CV and DCI should not be considered as separate disease entities; rather, CV should be considered as part of a multifactorial process, currently termed DCI, that can occur over a specific time period after SAH [13,14,19]. Several factors are discussed in the onset and manifestation of DCI, including vascular dysfunction, cortical spreading depolarization, and inflammatory processes within the CNS and cerebrospinal fluid (CSF), including the disruption of the blood–brain barrier (BBB) [14]. To further reduce morbidity and mortality after SAH, a better understanding of the pathophysiology of DCI and the inflammatory processes involved is essential [20,21], because inflammation is a critical component of the secondary or delayed injury cascade that follows SAH [22,23].

Inflammation is increasingly recognized as a contributor to DCI [14,24] and CV [25,26], and it seems to affect the outcome after SAH [23,27,28]. In this regard, pharmacological attempts to treat CV and DCI on the one hand improved CV but did not change the occurrence of DCI and thus the outcome (endothelin-1a antagonists). On the other hand, the outcome could be improved by administering calcium channel blockers, even without a direct improvement in CV or DCI [29,30,31,32]. A significant cellular component is also known from other inflammatory CNS diseases, in which B lymphocytes appear in low numbers but T lymphocytes and natural killer (NK) cells appear in significantly higher numbers and have more activity [33,34,35]. Pro-inflammatory and anti-inflammatory cells such as microglia, astrocytes, oligodendrocyte precursor cells, monocytes/macrophages, neutrophils, and especially NK cells and regulatory T (Treg) cells play a role in early and delayed cerebral injury processes after SAH and in the pathophysiology of DCI [23,32,36,37,38]. However, there is still little knowledge about the role of the cellular immune system in SAH. This is partly due to inadequate methods, as it is still very difficult to reliably distinguish the surface markers of, e.g., infiltrated monocytes, from activated microglia and perivascular macrophages in the CNS [32]. Cell-mediated inflammation can be caused by innate and adaptive immune cells. Analyses of adaptive immune cells (T cell and B cell subsets) in CSF and peripheral blood (PB) in patients with SAH are rare [39,40], and the inflammatory function of cell subsets like Treg cells [41,42,43,44] and NK cells [42,45,46] after human SAH is sparsely investigated [47]. Assuming that inflammation is a major contributor to CV, DCI, and thus to the associated outcome, it is important to identify the drivers of inflammatory processes, to determine whether an imbalance between self-tolerance and non-pathogenic reactivity is the cause (i.e., failure to tolerance) and, eventually, to develop therapeutic options [48].

The up- and downregulation of T cells or NK cells are controversially discussed in the literature. On the one hand, the downregulation of CD3^+^, CD4^+^, CD8^+^ T cells, NK cells, and Treg cells is associated with a poor outcome [49,50]. Other studies reported an increased activation of Treg cells in the advanced stage of DCI [37,51]. Since Treg cells can effectively influence the inflammatory effect of other T cells [52], a differentiated investigation of the two cell subpopulations in the outcome after SAH appears rational. A better understanding of cell-mediated inflammation in the occurrence of DCI and the outcome after SAH could eventually lead to a better assessment of patients at risk and the earlier initiation of specific therapies [23]. Therefore, this systematic review and meta-analysis aims to summarize and clarify the importance of cell-mediated inflammation in the outcome after SAH. Using relevant patient studies, this review further aims to answer three questions: 1. Does the activation of NK cells in PB (serum or plasma) or CSF have a negative influence on the functional outcome (favorable outcome according to mRS 0–2)? 2. Does the activation of Treg cells in PB (serum or plasma) or CSF have a negative influence on the functional outcome (favorable outcome according to mRS 0–2)? 3. Does the ratio of NK to Treg cells in PB (serum or plasma) or CSF have a negative influence on the functional outcome (favorable outcome according to mRS 0–2)? The added scientific value lies in the pooling of results in a field that is still very limited and challenging due to costly and complex detection methods. However, as a rapidly growing research field, corresponding investigations may reveal promising diagnostic and therapeutic options.

## 2. Results

### 2.1. Study Selection

**Figure 1:**
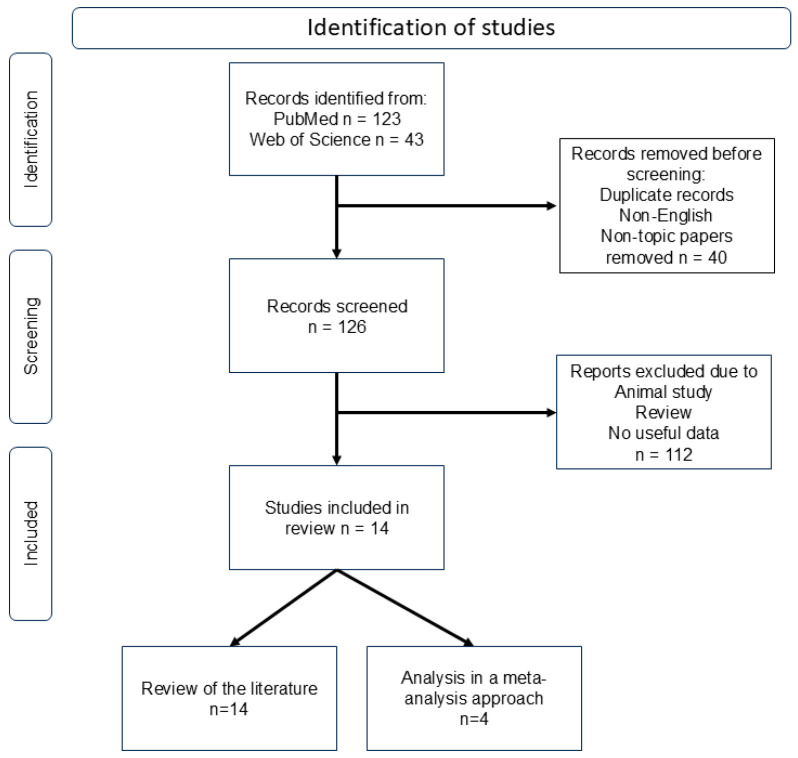
Identification, screening, and inclusion of studies. Abbreviation: n = number.

166 studies were identified from the scientific databases and literature search. The screening of titles, abstracts, and full content reduced the number to 14 studies related to inflammation, NK cells, and Treg cells in CSF and/or blood after aneurysmal subarachnoid hemorrhage in humans (Figure 1, Table 1) [37,39,40,42,45,46,53,54,55,56,57,58,59,60]. Only six studies contained detailed information about the relationship between NK cells or T cells and the 6-month outcome and mortality [37,39,45,46,58,60]. Of these, four were included in the meta-analysis (Table 2). The others served as data sources for the literature review.

### 2.2. Risk of Bias

Due to the fact that a low number of publications are published to this topic, the overall risk of bias was low. The results for the studies are given in Supplementary Materials S1.

### 2.3. Participants and Characteristics of Studies

The absolute number of patients included in this review was 411. The mean female percentage was 60.5% (SD: 10.6). The mean age was 53.4 (SD: 4.06) years. According to the available data (the Hunt and Hess score in seven studies and WFNS score in six studies), the median Hunt and Hess score was 4 (range: 3–5) and the WFNS score was 3 (range: 1–5). The Median Fisher’s grade was 4 (range: 3–4). The occurrence of DCI was reported in six studies with a rate of 20–100%. CV occurred in 39% to 80% of studies (seven studies). A favorable outcome was reported in five studies (0% to 75%), with a favorable outcome being defined as mRS 0–2. Mortality rates were reported in a range of 7% to 58% (six studies). However, the period of clinical follow-up differed. Table 1 shows the patients’ characteristics, treatment modalities, specimen, method, and outcome.

There was no standardized time course for cell analysis in all studies. Timepoints for analysis differentiated between several days and every day in a 14-day period (Table 1). Not all studies provided data from healthy controls. In most of the studies, cell analysis included fluorescence-activated cell sorting, or FACS (10 studies). Further methods are described, such as cytokine and chemokine analytics (enzyme-linked immunosorbent assay, ELISA), microscopic cell counting, polymerase chain reaction (PCR), chromatography, and microarrays. The analyzed cell types are leukocytes including monocytes and neutrophils, as well as more differentiated lymphocytes including T cells (Table 1).

**Table 1:** Patient characteristics and analysis.

Publication	Number of Patients	Mean Age (SD or Range)	Rate of Female Patients (%)	Hunt and Hess	WFNS Score	Fisher’s Grade	Surgical Treatment Strategy (%)	Rate of DCI (%)	Rate of Vasospasm (%)	Favourable Outcome After 6 Months (mRS)	Mortality Rate (%)	Specimen	Timepoint of Assessment	Method	Cell Type
**Literature review (330 patients)**															
Czlonkowska et al., 1979 Poland Prospective Control Group [53]	9	54 (21–70)	No information									PB	24–48 after admission	Cell counting	Leucocytes
Chrapusta et al., 2000 Poland Prospective Control group [54]	29	49 (12)	45	No information			100	No information	45	No information		PB	No Information	FACS Prolifeartion ECM assay	PBMC T cells
McMahon et al., 2013 United Kingdom Prospective Control group [56]	149	50 (23–78)	67	No information	1 (1–5)	3 (1–4)	10	31	No information			PB	Day 1–13	Blood count analysis, erythrocytes sedi-mentation rate, biochemical profile, ELISA	Leucocytes
Pera et al., 2013 Poland Prospective Control group [55]	43/15	55 (14/13)	51	3 (1) /4 (1)	No information						33	PB	Day 0–1,2–3, ≥4	Micro Array, PCR	PBMC
Moraes et al., 2015 Uruguay Prospective Control group [40]	12	49 (34–67)	67	5 (2—5)	No information	3 (1–4)	50	No information	42	No information	58	CSF and PB	Day 1–6	FACS Cell count	Leucocytes
Zhou et al., 2017 China Prospective No controls [45]	27	58 (9)	59	No information	2 (1–5)	4 (2–5)	100	No information		63	30	PB	Day 0,1,3,6	FACS, ELISA	Leucocytes
Roa et al., 2020 USA Propective Control group [42]	13	56 (14)	62	No information	4 (1–2,4–5)	4 (1,3–4)	0	No information	39	No information	8	CSF and PB	Day 0–1, 2–4, 5–9, ≥10	FACS, ELISA, Cell Count, IFI	NK cells T cells
Kim et al., 2020 Korea Recruitment from another prospective database No controls [57]	5	56 (3)	80	4 (3–4)	No information	4 (3–4)	0	100	No Information			PB	Day 0, 5, 7–9	HTS, PCS	T cells
Revilla-Gonzalez et al., 2023 Spain Prospective Control group [59]	28	57 (2)	68	3 (2–4)	2.5 (2–4)	4 (4–4)	No Information		46	No Information		PB	Day 0, 5	FACS, Adhesion assay	Leucocytes
Grossini et al., 2023 [58]	18	55 (48–71)	56	4 (3–5)	4.5 (4–5)	3.5 (3–4)	28	No information	44	No information	No information	PB	Day 0,1,7	Ultracentrifugation FACS	Extracellular Vesicles T cells
**Meta-Analysis (81 patients)**															
Mathiesen et al., 1990 Sweden Prospective [60]	8	47 (14)	63	No information			100	63	No information	75	25	CSF and PB	Day 1,3,6,9	Cell count Liquid CHG FACS	Leucocytes Erythrocytes
Spitzer et al., 2017 Germany Prospective No controls [46]	15	51 (9)	60	No information	3 (1–4)	3 (2–4)	34	53	80	47	7	CSF and PB	Day 1–14	FACS	NK cells
Mohme et al., 2019 Germany Prospective and retrospective cohort Retrospective controls [39]	25	58 (13)	68	3 (1–5)	No information	4 (2–4)	16	36	No information	52	No information	CSF and PB	Day 1–3, 4/5, 6/7, 8/9, 6–12	FACS, ELISA	Leucocytes T cells
Chaudhry et al., 2021 [37]	15	53 (12)	40	4 (I–III = 6 IV–V = 9)	No information	3 (3–4)	40	20	60	40	No information	PB	Day 1–3, 7–9	FACS	T cells
Total	411	53.4 (4.1)	60.5 (10.6)	4 (3–5)	3 (1–5)	4 (3–4)	43 (40)	51 (29)	51 (15)	46 (26)	27 (19)				

Abbreviations: SD = Standard deviation; WFNS = World Federation of Neurosurgical Societies Score; DCI = Delayed cerebral ischemia; mRS = modified Rankin Scale; FACS = Fluorescence-activated cell sorting; ELISA = Enzyme-linked immunosorbent assay; HTS = High-throughput sequencing; PCR = Polymerase chain reaction; CHG = Chromatography; NK cells = Natural killer cells; PB = Peripheral blood cells; CSF = Cerebrospinal fluid; ECM = Extra-cellular matrix; PBMCs = Peripheral blood mononuclear cells; IFI = Immunofluorescence imaging.

### 2.4. Favorable Outcomes and Mortality in Relation to Natural Killer Cells and Regulatory T Cells

**Figure 2:**
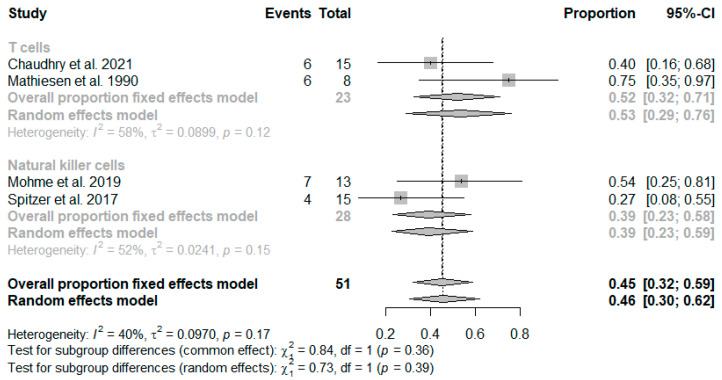
Forest plot illustrating the favorable outcomes (events) at six months based on the meta-analysis, presented in absolute (**left**) and proportional terms (**right**) sorted by cell type [37,39,46,60].

The studies included in the meta-analysis report favorable outcomes after 6 months at a frequency of 45% (CI: 0.32–0.59) (Table 2). However, in studies that considered T cells versus NK cells, there are differences in the occurrence of a favorable outcome after 6 months. Studies using the T cell approach reveal a favorable outcome more frequently than studies using NK cells as a readout (Figure 2).

**Figure 3:**
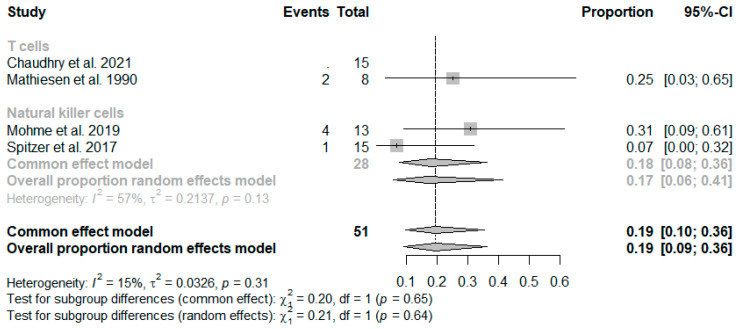
Forest plot illustrating mortality (events) based on the meta-analysis, presented in absolute (**left**) and proportional terms (**right**) sorted by cell type [37,39,46,60].

In terms of mortality, which averaged 19% (CI: 0.1–0.36), no clear distinction could be made based on the analyzed cell type (Figure 3). Importantly, studies exploring the relationship between NK cells and Treg cells in relation to outcomes and mortality were not included in the meta-analysis due to insufficient data.

**Table 2:** Characteristics of studies included in the meta-analysis.

Publication	Topic	No. of Patients	Effect	Definition of Effect	No. of Patients with Effect	Specimen	No. of Patients with Activation of Cells	No. of Patients with Activated Cells and Effect	Ratio of Patients with Effect and Activation	Higher Values in CSF than in Blood (Serum or Plasma)	Patients with Favorable Outcome	Patients with Cell Activation and Favorable Outcome	Patients with Fatal Outcome	Patients with Cell Activation and Fatal Outcome (Mortality Rate)
Mathiesen et al., 1990 [60]	Activation of T cells	8	DCI	clinically	5 of 8 (63%)	Serum	2 of 8 (25%)	2	5:2	4 of 8 (50%)	6 (75%)	2	2 (25%)	0
						CSF	6 of 8 (75%)	4	5:4			4		2
Spitzer et al., 2017 [46]	Activation of NK cells	15	CV	TCD radiologically	12 (80%)	Serum	15 (100%)	NA	NA	NA	4 (27%)	NA	1 (8%)	NA
						CSF		12	12:12			3		1
			DCI	clinically radiologically	8 (53%)	Serum		NA	NA			NA		NA
						CSF		8	8:8			3		1
Mohme et al., 2019 [39]	Activation of T cells	17	DCI	clinically radiologically	8 (62%)	Serum	NA	8	8:17	Yes (No specific information)	NA	NA	NA	NA
						CSF	NA							
	Activation of NK cells	13				CSF	13 of 13 (100%)				7 (54%)	7 (100%)	4	NA
Chaudhry et al., 2021 [37]	Activation of T cells (CD4+ T-cells, Treg cells)	15	CV	clinically TCD radiologically	9 (60%)	Serum	T reg 15 (100%)	9	9:9	NA	6	NA	NA	NA
			DCI		10 (67%)			0	0:10	NA				
								Not specified						

CV = Cerebral vasospasm; DCI = Delayed cerebral ischemia; NK cells = Natural killer cells.

*Natural killer cells in CSF after aneurysmal subarachnoid hemorrhage*

Spitzer et al. observed a greater increase in activated NK cells in CSF in patients with severe CV. The highest mean cell count was found at day 6, with the cell count decreasing afterward. In contrast, Mohme et al. found a decrease in NK cells in CSF at the late timepoint on days 6–12 compared to the early timepoint on days 1–4. A favorable outcome was seen in patients with a lower number of NK cells rather than an unfavorable outcome [39,46]. Similar findings were reported by Roa et al. They could demonstrate an increase in NK cells in the acute phase after SAH in the CSF of patients with CV and a progressive decline in the following days [42].

*Natural killer cells in peripheral blood after aneurysmal subarachnoid hemorrhage*

In PB, no significant difference in NK cell counts was observed between early and late timepoints following SAH [39]. Moraes et al. reported a lower percentage of NK cells in patients with SAH compared to controls during the first 3 days post-hemorrhage [40]. Conversely, Zhou et al. noted an increase in NK cell counts after surgical clipping in patients with SAH, with higher NK cell levels associated with favorable outcomes [45].

*Regulatory T cells in CSF after aneurysmal subarachnoid hemorrhage*

Treg cells increased in CSF after SAH within the first 9 days, according to a study by Mohme et al. [39]. This was confirmed by Chaudry et al. [37].

*Regulatory T cells in peripheral blood after aneurysmal subarachnoid hemorrhage*

In contrast, Treg cells declined in PB over time after SAH [39]. Zhou et al. reported higher Treg cell levels in patients with favorable outcomes, noting significant increases during both early and delayed brain injury phases compared to controls [45]. Interestingly, the Th17/Treg cell ratio was significantly lower in patients with SAH during both phases compared to controls, suggesting an imbalance in immune regulation [37].

*Natural killer cells, regulatory T cells, and their role in vasospasm, delayed cerebral ischemia, and outcome after aneurysmal subarachnoid hemorrhage*

The incidence of CV was documented in seven studies, while six studies reported on DCI. Detailed outcome data at 6 months were available from five studies, with mortality information included in six studies (Table 1). These studies varied in patient numbers, mean age, and follow-up duration. Unfortunately, none of the studies provided detailed data on the dynamic relationship between NK cells and Treg cells over time concerning the development of CV, DCI, or outcomes. Increased NK cell activity was associated with the presence of CV and DCI [39,46]. Conversely, reduced Treg cell activity was linked to the occurrence of DCI [37].

## 3. Discussion

Advances in surgical, endovascular, and intensive care therapy including imaging have improved results in the treatment of SAH [61,62,63]. Nevertheless, the pathophysiology of SAH with early and delayed brain injury or CV and DCI remain incompletely understood [14,64]. Knowledge about immunological and inflammatory involvement in delayed brain injury, including the development of CV and DCI, is constantly growing [23,65,66]. The immune response after SAH, especially the interplay between innate and adaptive immunity, plays a crucial role [23]. CSF leucocytes peak between the third and sixth day after SAH [67,68]. Lymphocytes peak in CSF around 17 days after SAH [69]. It should be noted that intrathecal cell-mediated inflammation can differ significantly from extrathecal processes, so that, for example, macrophage activity is not visible in peripheral blood [39]. Our literature review revealed that the involvement of NK and Treg cells in the development of CV and DCI is evident as well as their potential influence on clinical outcomes.

However, the current body of research remains limited in scope and depth. It remains unclear whether the effects involving NK cells and Treg cells are epiphenomena or are actually the expression of an underlying immunological process. In particular, their interplay has not been sufficiently investigated. In the following review, we aim to illustrate the significance of NK cells and Treg cells for the development of CV and DCI using various important components of delayed brain injury according to SAH.

### 3.1. Pathophysiology of Blood–Brain Barrier Breakdown After Aneurysmal Subarachnoid Hemorrhage

The BBB is a dynamic structure composed of endothelial cells, astrocytes, microglia, and pericytes, connected by tight, adherent, and gap junctions. It regulates molecular and cellular transport, ensuring CNS homeostasis [70,71,72,73,74,75]. SAH induces severe disruptions to the BBB, marked by the breakdown of tight junctions, increased permeability, and infiltration of immune cells, including neutrophils and macrophages. This damage is exacerbated by inflammatory cytokines such as TNF-alpha, IL-1, and IL-6, which further degrade BBB integrity [70,75,76].

Following SAH, the influx of blood into the CSF triggers a cascade of events: elevated intracranial pressure (ICP), reduced cerebral perfusion pressure, and ischemia. These conditions lead to cytotoxic edema, endothelial apoptosis, and vasogenic edema, all contributing to BBB collapse. Degradation products like oxyhemoglobin and reactive oxygen species exacerbate this process, reaching a peak between days 5 and 7 post-hemorrhage. This coincides with the onset of delayed cerebral ischemia (DCI) and vasospasm [25,28,64,77,78,79,80,81,82,83,84,85]. The BBB’s breakdown facilitates the infiltration of peripheral immune cells, including Treg cells and NK cells [86].

NK cells contribute to neuronal damage by releasing perforin and pro-inflammatory cytokines like IFN-gamma. These processes aggravate inflammation and tissue damage within the CNS [87,88].

Conversely, Treg cells play a crucial role in modulating this inflammatory response. By secreting anti-inflammatory cytokines such as IL-10 and TGF-beta, Treg cells help mitigate BBB damage and restore immune balance [36].

### 3.2. Pathophysiology of Delayed Cerebral Injury in Aneurysmal Subarachnoid Hemorrhage

DCI results from an imbalance between cerebral perfusion and the metabolic demands of various cells, particularly neurons and glial cells. This imbalance ultimately leads to inadequate oxygen and nutrient supply to these cells, with the most detrimental effects being on their survival and function [14]. A widely used clinical definition of DCI is “The occurrence of focal neurological impairment (such as hemiparesis, aphasia, apraxia, hemianopia, or neglect), or a decrease of at least 2 points on the Glasgow Coma Scale (either on the total score or on one of its individual components [eye, motor on either side, verbal]). This should last for at least 1 h, is not apparent immediately after aneurysm occlusion, and cannot be attributed to other causes by means of clinical assessment, CT or MRI scanning of the brain, and appropriate laboratory studies” [64,89]. Furthermore, it is seen as a result of several parallel and sequential molecular and cellular events within the first 24 to 72 h after SAH (early brain injury phase), with clinical consequences appearing from the 4th to 12th day (delayed brain injury phase) after hemorrhage. These events are the mentioned initial increase in ICP, the subsequent global ischemia, the various forms of cellular edema and cerebral edema mentioned earlier, the dysfunction and loss of function of the BBB, synaptic activation, the disruption of cerebrovascular autoregulation, the formation of microthrombi, the occurrence of spreading depolarizations, and cerebral thromboinflammation [14,90].

The transient global cerebral ischemia resulting from increased ICP leads to excessive activation of the sympathetic nervous system [78,79,80,81,91] and the stimulation of the endothelin-1 (ET-1) pathway, which is associated with a decreased cerebral blood flow [92,93]. This combination of factors exacerbates ischemia and further impairs cerebral perfusion.

The oxyhemoglobin released by lysed erythrocytes in the CSF has a vasoconstrictive effect on cerebral vessels in the DCI phase via the Rho/Rho-associated protein kinase (Rho/Rock) and protein kinase C (PKC) pathways [94,95]. In addition, oxyhemoglobin has a significant reducing effect on the production of the vasoconstrictive NO [26,96,97]. Oxyhemoglobin interacts with dimethylarginine, a nitric oxide synthase (NOS) inhibitor, other NOS enzymes, oxygen, and the generation of superoxides and peroxynitrites for the further vascular dysfunction included in the clinical picture of CV [96,97,98,99,100,101,102]. In the context of platelets entering the CSF, the NO–cyclic guanosine monophosphate (cGMP) pathway and the endothelial protease ADAMTS13, along with the von Willebrand factor and P-selectin pathways, play crucial roles under physiological conditions in regulating the prothrombotic and vasospastic properties of platelets. Disruptions in these processes can result in the formation of microthrombi and the occurrence of vasospasms in intraparenchymal arterioles, further contributing to hypoxia, ischemia, and tissue damage [103,104,105,106,107,108,109,110,111,112,113,114,115,116,117,118]. This prothrombotic circumstance is exacerbated by the fact that the damaged glycocalyx does not have a physiological site of action for anticoagulant molecules such as anti-thrombin, the von Willebrand factor, and the tissue factor inhibitor pathway [117,119,120]. It remains an open question whether antithrombotic agents exert a beneficial effect on DCI after SAH [121,122].

The pro-inflammatory role of microglia, including toll-like receptor 4 (TLR4) activation, is under investigation [25,123,124,125,126]. After SAH, microglia may recruit peripheral myeloid and lymphoid cells, exacerbating neuronal injury through the release of cytokines like MIF and adhesion molecules such as ICAM-1 and VCAM-1. This contributes to cerebral vasculopathies, oxidative stress, and endothelial damage [14,64,127,128,129,130,131,132,133]. Increased K⁺ and hemoglobin from cell lysis, along with hypoperfusion and inflammation, disrupt neuronal signaling through membrane depolarization and Na⁺/K⁺ pump dysfunction. This leads to cellular damage, neuronal death, and reactive gliosis [64,134,135]. The collective effect of these inflammatory cells, pathways, and processes plays a critical role in the onset and severity of DCI and ultimately determines the individual outcome of a patient [14,25,136,137,138,139,140].

### 3.3. Pathophysiology of Inflammatory Processes After Aneurysmal Subarachnoid Hemorrhage Including Natural Killer Cells and Regulatory T Cells

In all acute brain injuries, inflammation plays a role in pathophysiological processes, and this also applies to SAH [24,66,86,141,142,143,144]. Most authors describe an early brain injury (EBI) phase within the first 72 h after hemorrhage. More rarely, an acute brain injury (ABI) phase is also recognized. This is usually followed by the delayed or chronic brain injury (DBI) phase [26,32]. Initial pathophysiological processes involving inflammation resulting in increased ICP, decreased brain perfusion, ischemia, the breakdown of the BBB, cerebral edema, CV, autophagy, apoptosis, necrosis, and necroptosis are associated with the EBI and ABI phases [145,146,147,148,149,150,151,152,153]. In the second phase, these processes are amplified one after the other, but also partly in parallel, and cellular and molecular repair processes occur, the extent of which is decisive for the outcome [149,154]. This is the phase in which CV and DCI mainly occur as a result of inflammatory processes, among others [26]. One challenge for studies is the differing accessibility of resident inflammatory cells in the brain, such as microglia, astrocytes, and oligodendrocytes, compared to peripheral immune cells like monocytes/macrophages and dendritic cells. This difference in accessibility complicates the study of their distinct roles in the inflammatory response, as resident cells are activated earlier, while peripheral cells are recruited later, contributing to the complexity and progression of neuroinflammation after SAH [32]. Inflammatory processes in the CNS are not only represented by immune cells recruited from outside the BBB. Rather, they are represented by a variety of cells that are recruited from the blood, are activated intrathecally, and partially leave the CNS again after their activation [33,141,155]. The function and influence of NK cells and Treg cells after SAH is a rather new research field [156]. After SAH, there is an initial downregulation of CDD3^+^, CD4^+^, and CD8^+^ T cells, NK cells, and Treg cells in the EBI phase [50]. In the DBI phase after SAH, however, an increased activity of NK and Treg cells in the CSF is also seen [23,37,49]. The exact influence of NK cells on Treg cells on the one hand, but also of NK cells and Treg cells on the occurrence of CV and DCI as well as on the outcome of patients, is still poorly investigated and unclear. There are only a few patient studies on the involvement of NK cells and Treg cells. The course of activation in the CSF is controversial. An increase in the CSF on days 1 to 6 is reported for NK cells. This then leads to a decrease in concentration or activity in the CSF [46]. CD6 and CD56 (cytotoxic)-expressing NK cells appear to have a temporal connection with the development of clinically detectable CV [46]. Of note, an increase in CD8+-expressing T cells between days 3 and 8 after SAH is associated with the development of DCI [60].

NK cells, members of the group 1 innate lymphocytes (ILC1), are considered effector lymphocytes of the innate immune system, with key roles in microbial defense and tumor surveillance [157]. They are able to distinguish stressed cells from healthy cells and lyse them through a process that has not yet been fully decoded. Various cell ligands and pathways such as human ULBP, MIC molecules, and toll-like receptors (TLRs) play a role in this process [157]. In humans, NK cells can be differentiated into CD56^dim^ and CD56^bright^.

CD56^dim^ CD16^+^ NK cells constitute the majority (about 90%) of NK cells in the blood and spleen. These cells are highly cytotoxic and express perforin, which allows them to kill target cells, such as infected or tumor cells. CD56^bright^ CD16^−^ NK cells are less abundant and are predominantly found in lymph nodes and tonsils. These cells are more involved in cytokine production and play a key role in modulating the immune response rather than directly killing target cells [155,157,158]. NK CD56^bright^ cells predominate in the CSF [142,159,160]. The cytotoxic function of NK cells, which are non-antigen-specific immune cells, is tightly regulated by a balance between inhibitory and stimulatory receptor activity on their surfaces [66]. This regulation is further influenced by cytokines such as interferons (IFNs), interleukins (IL-2, IL-12, IL-15, IL-18), and transforming growth factor-beta (TGF-β). Additionally, interactions with other immune cells, including T cells, dendritic cells, and macrophages, play a crucial role in modulating NK cell activity. This intricate control ensures that NK cells effectively target infected or transformed cells while minimizing damage to healthy tissues [157,161,162]. MHC class I molecules on healthy cells send inactivating signals to NK cells. In contrast, messenger substances such as retinoic acid early-inducible gene (RAE-1)-encoded proteins, which are expressed on the surface of pathogenic virus-infected or tumor cells, serve as activation signals for NK. Stimulation is followed by the cytotoxic effect via contact-dependent pathways and the production of further pro-inflammatory cytokines [144]. NK cells can also exert regulatory or cytotoxic effects on other cell types, including T cells, B cells, and endothelial cells [88,157,163,164,165]. Some authors classify NK cells based on the expression of CD27, a member of the tumor necrosis factor receptor family, to distinguish different functional subsets. Tolerant NK cells, characterized by a CD56^bright^ phenotype with CD27^−^ and CD11b^−^ markers, are thought to play a role in maintaining immune tolerance and preventing excessive immune responses. In contrast, cytotoxic NK cells, which exhibit a CD56^dim^ phenotype with CD11b^+^ and CD27^−^ markers, are highly cytotoxic and primarily involved in the direct killing of infected or tumor cells. Meanwhile, regulatory NK cells, identified as CD56^bright^ with CD27^+^ markers, represent approximately 6% of all NK cells in the blood and primarily perform immunoregulatory functions, such as cytokine production and the modulation of other immune cells [166,167,168,169,170]. The interest in the function of NK cells after SAH stems from two key observations. First, NK cells, originally understood as effectors in microbial and neoplastic defense, exhibit altered activity following SAH despite the absence of microbial or neoplastic triggers. Second, their interaction with T cells demonstrates a temporal relationship with clinical outcomes, suggesting a significant role in post-SAH pathophysiology. These considerations are particularly relevant because NK cells can suppress T cell activation, either by killing antigen-presenting cells or by secreting immunosuppressive cytokines, thereby influencing the immune response and its consequences after SAH [144]. When the BBB is disrupted, NK cells exhibit a marked increase in activity, contributing significantly to inflammatory processes in the central nervous system [86]. How exactly NK cells migrate to the brain and interact with neurons despite the BBB has not yet been clarified in detail [86,142]. NK cells utilize adhesion molecules such as VLA-4 on their surface and VCAM-1 expressed on endothelial cells of the BBB. This interaction supports their initial attachment and movement along the endothelial cells [171]. Chemokines secreted during neuroinflammation or in pathological conditions create a gradient that guides NK cells. Specific chemokines bind to corresponding chemokine receptors on NK cells, directing their migration toward areas of higher chemokine concentrations [172].

In contrast to the inflammatory role of NK, Treg cells perform diverse and complex immunomodulatory functions. They can produce both pro-inflammatory cytokines, such as IL-17, and anti-inflammatory cytokines, including IL-10, IL-35, and TGF-β. Treg cells influence the proliferation and function of various immune cell types, including other T cells, B cells, NK cells, monocytes, macrophages, and dendritic cells. Furthermore, Treg cells exhibit cytolytic abilities, allowing them to directly eliminate target cells under certain conditions [48,52,173,174,175,176,177,178,179,180,181,182,183,184,185,186,187,188]. Treg cells are also present in the brain and meninges, where they play a critical role in modulating immune responses. In these locations, Treg cells exert a suppressive effect on both pro-inflammatory T cell subsets, such as those producing TNFα and IFN-γ, and anti-inflammatory T cell subsets [23,66,182,189,190,191,192,193,194]. In terms of physiological status, CD4^+^ T cells make up the majority in the CSF, along with granulocytes, B cells, and NK cells [195]. Under physiological conditions, cells of the adaptive immune system, particularly Treg cells and other immune cell subsets, generally predominate over the innate immune system in the CNS. These adaptive immune cells help protect the BBB by suppressing the production of matrix metalloproteinase-9 (MMP-9), an enzyme that contributes to BBB breakdown and neuronal damage. Additionally, adaptive immune cells are involved in downregulating neuroinflammation [196].

The role of Treg cells in the CNS has been relatively underexplored until recently. However, emerging studies suggest that Treg cells in the CNS play a neuroprotective role. This protective function is thought to be mediated through interactions with various cell types, including neurons, microglia, astrocytes, endothelial cells, and oligodendrocytes. Notably, Treg cells may exert their effects through the CCL22 and CCR4 pathways, which are involved in the recruitment and signaling of these cells within the CNS [183,192,193,197,198,199,200,201]. Their role after acute vascular events of the brain is attributed to the regulation of pro-inflammatory cytokines and thus the progression of tissue damage, the modulation of the recruitment of further peripheral immune cells such as lymphocytes, and the activation of the brain’s own resident immune cells such as microglia [202]. In a mouse model of ischemic stroke, Treg cells have been shown to play a crucial role in modulating the immune response and promoting recovery. Treg cells reduce the production of the pro-inflammatory cytokine IL-6 through the secretion of amphiregulin, which helps limit the overall inflammatory response. Additionally, Treg cells enhance microglia-mediated repair mechanisms by increasing osteopontin expression, a molecule involved in tissue repair and remodeling. Moreover, Treg cells can reduce astrocyte activity through the inhibition of STAT3 phosphorylation, a signaling pathway that typically promotes astrocyte activation in response to injury [183,193,203].

**Figure 4:**
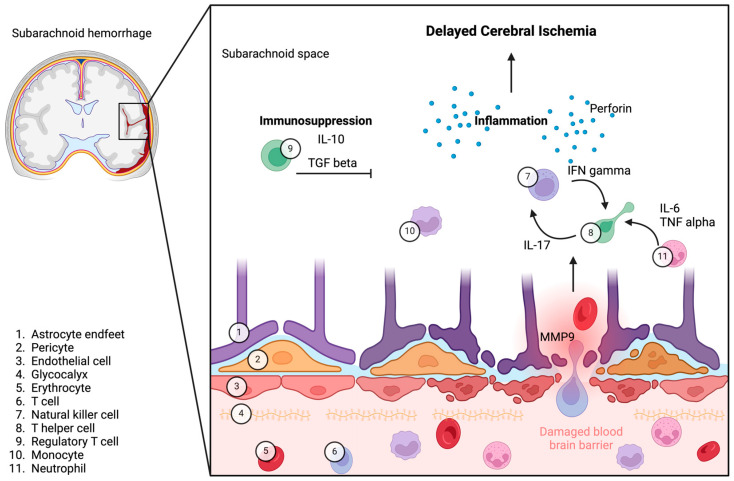
A proposed hypothesis for the pathophysiological role of neuroinflammation in the development of DCI in patients with SAH with emphasis on the roles of Treg cells and NK cells. The rupture of an aneurysm releases blood and leukocytes into the CSF, triggering an inflammatory cascade. The dysregulation of Treg cells and activation of NK cells contribute to this process, exacerbating the inflammatory cascade. We propose that this inflammatory response may play a crucial role in the development of DCI in patients with SAH.

Treg cells play a protective role in maintaining the integrity of the BBB [183,196]. As previously mentioned, neutrophils and their production of MMP-9 are detrimental to the BBB, contributing to its breakdown. However, through direct contact with neutrophils, Treg cells can reduce MMP-9 expression via the PD-L1 pathway, thereby protecting the BBB from this destructive effect [204,205,206]. In addition, NK cells recruit neutrophils after intracerebral hemorrhage, leading to a destructive effect on the BBB [207]. Of note, Treg cells can inhibit NK cells and suppress IL-17-mediated disruption of the BBB (Figure 4) [208,209,210,211]. Furthermore, by supporting oligodendrocytes, Treg cells can strengthen the regeneration processes in the CNS [212]. The number of Treg cells in peripheral blood can be increased by administering IL-2 [213], and this can lead to a smaller infarct volume [214,215]. A lack of Treg cells or their immunomodulatory function can therefore result in an uncontrolled inflammatory process culminating in severe structural and functional damage to the brain, as described above [191,202,216].

Treg cells regulate NK cell functions by controlling their recruitment, proliferation, cytotoxicity, and cytokine production. This helps limit excessive immune responses and tissue damage in neuro-inflammatory conditions like stroke or hemorrhage [176,217,218,219]. However, the influences of NK cells on the function of Treg cells must also be taken into account [164,217,220,221]. In many respects, these cells represent the target of immunomodulatory therapy approaches [218].

### 3.4. Emerging Therapeutic Approaches

Fingolimod, an immunomodulatory agent primarily used for multiple sclerosis, has been investigated for its potential benefits in treating DCI following SAH. In a rat model of SAH, fingolimod administration was associated with reduced leukocyte adhesion to pial venules, the preservation of arteriolar dilation, and improved neurological outcomes [222]. Fingolimod regulated the inflammatory response after SAH in a mouse model and improved neurological recovery. After 3 days of treatment with fingolimod, Treg cells were significantly increased and NK cells were downregulated in the brain tissue [223]. These findings suggest that fingolimod may mitigate the neuroinflammation and vascular dysfunction associated with DCI after SAH.

The potential neuroprotective role of Treg cells is being increasingly studied as a treatment option. Treg cells exhibit anti-inflammatory properties that provide neuroprotection in the ischemic brain, contributing to reduced infarct size and enhanced neuronal recovery [224].

For instance, adoptive Treg cell therapy in rodent models of ischemic stroke demonstrated significant reductions in brain infarct size and sustained neurological improvements. This neuroprotection was associated with decreased blood–brain barrier disruption, reduced cerebral inflammation, and limited infiltration of peripheral immune cells. Notably, Treg cells appeared to exert their protective effects without directly entering the brain parenchyma, instead modulating peripheral immune responses, such as suppressing neutrophil-derived matrix metallopeptidase-9 production, which is implicated in blood–brain barrier integrity [196].

In the context of SAH, Wang et al. conducted a study using a rat SAH model in which blood was injected twice into the cisterna magna, followed by the intravenous administration of Treg cells to the rats. The transfer of Treg cells significantly reduced SAH-induced brain edema and improved cerebral blood flow. Their research highlights that Treg cell adoptive transfer could attenuate SAH-induced cerebral inflammation by suppressing the activation of the TLR4/NF-κB signaling pathway, which is central to inflammatory responses [225]. Although these findings are promising, further research is necessary to validate the effectiveness and safety of Treg cell-based therapies for DCI post-SAH in both preclinical and clinical settings.

### 3.5. Limitations of Previous Research and Meta-Analysis of Natural Killer Cells and Regulatory T Cells After SAH

The absence of data from healthy control groups in two studies in the meta-analysis and five among the reviewed studies presents a significant limitation, as such data are crucial for establishing baseline immune responses and contextualizing pathological changes. Without a well-defined reference point, interpreting deviations in immune markers or cellular behaviors becomes more challenging.

The lack of a standardized time course for cell analysis across the studies introduces variability that can complicate comparisons and interpretations. Different timepoints can significantly impact observed NK cell and Treg cell dynamics, especially in processes such as inflammation or immune activation, which are highly time-dependent. This variability makes it difficult to discern whether differences are due to biological factors or the timing of measurements.

A notable concern for the methodologies utilized across the included studies, such as FACS and cytokine and chemokine arrays, is the lack of standardization across studies. Variability in sample preparation, antibody selection, gating strategies, and analytic platforms can introduce inconsistencies, potentially leading to challenges in replicability and cross-study comparisons.

While there is growing knowledge about NK cells and Treg cells in CSF and their serotonin responsiveness, distinguishing between resident intrathecal cells and those activated peripherally remains challenging. This difficulty complicates the accurate identification and characterization of NK and Treg cell activation in SAH using FACS, making it hard to differentiate between local and peripheral immune cell responses [191]. In order to achieve further improvements in outcome after SAH, it is necessary to address this research area in the future, e.g., within stroke research.

## 4. Materials and Methods

This systematic review and meta-analysis approach was based on an extensive literature search in accordance with the Preferred Reporting Items for Systematic Reviews and Meta-Analysis (PRISMA) guidelines [226]. As cohort studies and case–control studies were expected to be included in this review, the Newcastle–Ottawa scale was used to assess the quality of the methodology of the studies. This scale was used by two experienced neurosurgeons [227].

### 4.1. Search Strategy

The literature search was conducted using Medline/PubMed and the Web of Science as databases. For the Boolean search, the term “aneurysmal subarachnoid hemorrhage” was combined with the following keywords and phrases:

1. “t cells”;
2. “delayed cerebral ischemia” and “t cells”;
3. “vasospasm” and “t cells”;
4. “natural killer cells”;
5. “neuroinflammation” and “delayed cerebral ischemia”.

In total, 166 publications could be identified in different languages. A total of 40 reports had to be rejected because they represented duplicates or were not in the English language. The remaining 126 publications were tested for inclusion through the reading of the abstract or the full content.

An additional 112 publications were excluded from analysis as they were either animal studies, reviews, or lacked essential information on patient characteristics, specimen details, or analysis methods. The remaining 14 publications were deemed relevant and subjected to further investigation (Figure 1). These studies provide insights into the effects and regulation (upregulation and downregulation) of NK cells and T cells. However, only four of these studies included detailed data on 6-month outcomes and mortality. These four studies were incorporated into the meta-analysis, presented using forest plots. The analysis adhered to the PRISMA (Preferred Reporting Items for Systematic Reviews and Meta-Analyses) guidelines [228].

### 4.2. Eligibility Criteria

The studies included represent retrievable English scientific reports related to the CSF and/or blood analysis of human patients who suffered from SAH and underwent in-hospital treatment. Further inclusion criteria involved the detailed reporting of cell analysis methods in the context of described DCI, CV, or detected neuroinflammation. Cell analysis had to include T cells and/or NK cells in CSF and/or blood. Inclusion criteria were further expanded to include patient age, gender, clinical grade (Hunt and Hess score [229], World Federation of Neurosurgical Societies score [230,231], Fisher’s score [232,233]), the method of aneurysm sealing (surgical or endovascular), and the occurrence of CV and DCI. For the meta-analysis, the mortality rate and rate of favorable outcomes needed to be included. The outcome should have been standardized using either the Glasgow Outcome Scale or the modified Rankin Scale [234,235]. Studies and reports without the mentioned information were excluded.

### 4.3. Study Selection

Only full-text publications were screened for their titles, abstracts, and full content by two independent neurosurgeons. Any relevant publication was further screened for eligibility as mentioned above. There were no disagreements between the screening individuals.

### 4.4. Risk of Bias Assessment

For the purpose of a risk of bias assessment, the Joanna Briggs Institute (JBI) criteria appraisal checklist was used [236,237]. To ensure transparency, it needs to be mentioned that the authors themselves conduct research in this field, that a publication by the authors was included in this review, and that the authors are therefore not completely independent.

### 4.5. Data Extraction and Analysis

For evaluation, data about patients’ characteristics including demographic data (sex and age), clinical presentation (Hunt and Hess score [229], World Federation of Neurosurgical Societies score [230,231]), imaging data (Fisher’s score [232,233]), the aneurysm treatment strategy (surgical or endovascular), the occurrence of neurovascular complications (CV and/or DCI), and the outcome (modified Rankin Scale [238,239], favorable outcome 0–2; mortality rate) were recorded in a database. Furthermore, data about the specimen (CSF and/or PB) and the time course of sample collection (daily, every two days, weekly), as well as the analyzed cell type (leucocytes, lymphocytes) and analysis method (FACS, ELISA, PCR), were recorded. The main features of the meta-analysis approach were the outcome and mortality related to inflammatory cells and CV or DCI after SAH.

### 4.6. Statistical Analysis

In order to combine results from all the identified studies regarding favorable outcomes after six months in hospital and overall mortality, a meta-analytic approach was used. In particular, the reported overall proportions from the included single-arm studies were combined using the inverse variance method, which is available in the R package “meta” version 4.3.2. Study heterogeneity was assessed using the I^2^ measure, leading to a fixed effects combination model in the case of I^2^ < 50%, and to a random effects combination model otherwise. For the statistical interpretation of the overall proportions calculated for each procedure separately, 95% confidence intervals (CIs) were used, where non-overlapping CIs indicated statistical significance (*p* < 0.05) [240].

## 5. Conclusions

This review highlights the significant yet complex roles of NK cells and Treg cells in the pathophysiology and outcomes of aneurysmal SAH. Studies demonstrate that NK cell activity increases in CSF during the acute phase of SAH, particularly in patients with CV, but declines over time. Higher NK cell activity is associated with CV and DCI, suggesting a link between heightened innate immune responses and adverse outcomes. Conversely, regulatory T cells increase in CSF but decline in peripheral blood, with higher Treg cell levels correlating with favorable outcomes. The imbalance in the Th17/Treg cell ratio further emphasizes the role of immune dysregulation in the progression of SAH complications.

However, inconsistencies in methodologies, including variations in timepoints for cell analysis and the lack of data on the dynamic interplay between NK cells and Treg cells, limit the ability to draw definitive conclusions. Moreover, the absence of standardized approaches and insufficient inclusion of healthy controls hinder cross-study comparisons.

Future research should focus on standardizing protocols and conducting comprehensive longitudinal studies to better understand the interactions between NK cells and Treg cells. Such studies could elucidate their precise roles in the development of CV and DCI and overall patient outcomes. Both NK and Treg cells hold promise, not only as biomarkers for predicting severe disease progression but also as potential targets for innovative therapeutic interventions. Advancing our understanding of the underlying pathophysiological mechanisms could accelerate the development of effective treatments for inflammation following SAH. Further investigations into therapies such as fingolimod and Treg cell-based approaches may offer valuable insights and potentially improve outcomes for patients in the long run.

## Data Availability

The data analyzed in this systematic review are freely accessible in the medical databases. Further data requests can be made to the authors. The authors will provide available data or assist in finding them in the databases.

## Supplementary Materials

The following supporting information can be downloaded at https://www.mdpi.com/article/10.3390/ijms26031276/s1.
