# Supplementary material for: Regulatory T Cell- and Natural Killer Cell-Mediated Inflammation, Cerebral Vasospasm, and Delayed Cerebral Ischemia in Aneurysmal Subarachnoid Hemorrhage—A Systematic Review and Meta-Analysis Approach
Source: Int J Mol Sci. 2025 Feb 1;26(3):1276. doi: 10.3390/ijms26031276 (PMC11818301; doi:10.3390/ijms26031276)
Supplement: Supplementary file 1 [file ijms-26-01276-s001.zip › ijms-3392685-supplementary.pdf]

PRISMA 2020

Overview of PRISMA Alignment

Section and Topic	Item #	Checklist Item	Location in Manuscript
**TITLE**	1	Identify the report as a systematic review.	Title: "T-regulatory and Natural Killer Cell-mediated inflammation...a systematic review and meta-analysis approach"
**ABSTRACT**	2	See the PRISMA 2020 for Abstracts checklist.	Summarizes objectives, methodology, and key findings.
**INTRODUCTION**	3	Describe the rationale for the review in the context of existing knowledge.	The immune cell involvement in the pathophysiology of SAH with emphasis on T cells and NK cells.
**INTRODUCTION**	4	Provide an explicit statement of the objective(s) or question(s) the review addresses.	This systematic review and meta-analysis aim to summarize and clarify the importance of cell-mediated inflammation for the outcome after SAH. Using relevant patient studies, this review further aims to answer three questions: 1. Does activation of NK cells in PB (serum or plasma) or CSF have a negative influence on the functional outcome (favorable outcome according to mRS 0-2)? 2. Does activation of Treg cells in PB (serum or plasma) or CSF have a negative influence on the functional outcome (favorable outcome according to mRS 0-2)? 3. Does the ratio of NK to Treg cells in PB (serum or plasma) or CSF have a negative in-fluence on the functional outcome (favorable outcome according to mRS 0-2).
**METHODS**	5	Specify the inclusion and exclusion criteria for the review and how studies were grouped for synthesis.	Methods: Inclusion criteria detailed, including CSF/blood analysis, DCI, CV, and patient outcomes, see Eligibility Criteria in the manuscript.

**METHODS**	6	Specify all databases and other sources searched, with dates.	Methods "Medline/PubMed and Web of Science..." with search terms and language restrictions detailed. Qualifying date was June 30th 2024.
**METHODS**	7	Present the full search strategies for all databases, including filters/limits used.	Methods: Boolean search strategy described for SAH-related terms (e.g., "t cells", "vasospasm").
**METHODS**	8	Specify the methods for deciding whether a study met inclusion criteria.	Methods: See chapter Eligibility Criteria
**METHODS**	9	Specify data collection methods, including reviewer independence.	Methods: Data extraction by 2 independent reviewers, specific variables recorded (e.g., demographics, outcomes).
**METHODS**	10a	List and define all outcomes for which data were sought.	Methods: Outcomes include mRS, mortality, and inflammatory cell dynamics.
**METHODS**	10b	List and define all other variables sought (e.g., participant/intervention characteristics).	Methods: Includes patient demographics, clinical grading (Hunt & Hess), aneurysm treatment, CV/DCI occurrence.
**METHODS**	11	Specify methods to assess risk of bias.	Methods: "Joanna Briggs Institute criteria" applied, transparency about potential bias due to author publications.
**METHODS**	12	Specify effect measures for outcomes.	Methods: Effect measures include proportions with confidence intervals for meta-analysis using the R package.
**RESULTS**	16a	Describe the results of the search and selection process.	Results: "166 studies identified... reduced to 14 after screening (Figure 1)."
**RESULTS**	16b	Cite excluded studies and reasons for exclusion.	Results: "112 publications excluded due to being animal studies, reviews, or lacking key information."
**RESULTS**	17	Cite and describe characteristics of included studies.	Results: Study characteristics summarized in Table 1 (e.g., patient demographics, methods, outcomes).
**DISCUSSION**	23a	Provide interpretation of results in context of other evidence.	Discussion: Links findings to prior research, highlighting immune cell roles in SAH and complications like DCI.

**DISCUSSION**	23b	Discuss limitations of included evidence.	Discussion: Notes limited scope, inconsistent time points, and absence of control groups in some studies.
**DISCUSSION**	23c	Discuss limitations of review processes.	Discussion: Acknowledges methodological variability and limited sample sizes.
**DISCUSSION**	23d	Discuss implications for practice, policy, and future research.	Discussion: Calls for standardized protocols, detailed immune cell studies, and exploration of NK/Treg interactions.
**OTHER INFORMATION**	25	Describe sources of support and roles of funders.	Funding: "No external funding."
**OTHER INFORMATION**	26	Declare competing interests.	Conflicts of Interest: "Authors declare no conflicts of interest... Transparency about inclusion of own research."

This checklist aligns PRISMA 2020 items with the manuscript content to confirm adherence.
